# Population Genetic Structure of *Aphis gossypii* Glover (Hemiptera: Aphididae) in Korea

**DOI:** 10.3390/insects10100319

**Published:** 2019-09-26

**Authors:** Hwa Yeun Nam, Yujeong Park, Joon-Ho Lee

**Affiliations:** 1 Entomology Program, Department of Agricultural Biotechnology, Seoul National University, Seoul 08826, Korea; jessienam@snu.ac.kr (H.Y.N.); bravohpark@snu.ac.kr (Y.P.); 2 Research Institute of Agriculture and Life Sciences, Seoul National University, Seoul 08826, Korea

**Keywords:** *Aphis gossypii*, microsatellite, genetic structure, genetic diversity

## Abstract

*Aphis gossypii* Glover (Hemiptera: Aphididae) is a serious polyphagous agricultural pest worldwide. In the present study, we used eight microsatellite markers to investigate the genetic structure and diversity of *A. gossypii* populations in Korea. Samples were collected from 37 locations in Korea (18 populations in 2016, 14 populations in 2017, and five populations in 2018) from pepper plants. *A. gossypii* had low to moderate genetic diversity, and expected heterozygosity (*H*_E_) ranged from 0.354 to 0.719. A Mantel test of isolation by distance indicated no relationship between genetic structure and geographic distance among all populations (*r*^2^ = 0.0004, *p* = 0.370), suggesting high gene flow among populations in Korea. Populations of *A. gossypii* in Korea were divided into two distinct genetic clusters (Δ*K* = 2). In 2016 and 2017, the genetic clusters changed into opposite genetic structures within one year mostly in northwest and southeast parts of Korea. Possible relevance of study results was discussed. Chemical control, cyclical parthenogenesis, and immigrants from the exterior might have resulted in this low genetic diversity and opposite genetic clusters.

## 1. Introduction

The cotton–melon aphid, *Aphis gossypii* Glover (Hemiptera: Aphididae), is a worldwide polyphagous insect species, with a large ecological and host range [1]. *A. gossypii* is an important pest of many agricultural plants, including cotton, melon, potato, chili pepper, sweet pepper, and eggplant [2,3]. It causes significant damage to host plants by sapping, dripping honeydew, and transmitting viruses.

*A. gossypii* exhibits anholocyclic or holocyclic forms of life cycle [4]. Anholocyclic *A. gossypii* overwinters as nymphs or adults, while holocyclic *A. gossypii* shows a heteroecious or autoecious lifecycle. Heteroecious *A. gossypii* migrates from buckthorn as its primary host to herbaceous plants as its secondary host, where it reproduces asexually to produce numerous offspring in the spring. It then returns to the primary host in the fall to lay overwintering eggs [4]. On the contrary, autoecious *A. gossypii* does not require any secondary host. Only holocylic *A. gossypii* was found in several countries including Korea where harsh winter is common [5]. In Korea, *A. gossypii* hatches from eggs on its primary host in the beginning of April and reproduces for two to three generations before adults (apterous viviparous females) migrate to secondary hosts from May to June [6,7].

Understanding population genetic structure of *A. gossypii* could help to manage aphid populations by providing more reliable estimates of population dynamics and the risk of resistance genes arising [8]. The genetic structure of *A. gossypii* populations is influenced by various factors such as host plants, geographical barriers, insecticides, and dispersal ability [9,10]. Significant genetic differentiation and different population structure are observed for most aphid species because their gene flow is limited among populations due to their weak flying ability and reproductive characteristics [11]. Aphid is a parthenogenetic species with a high clonal diversity, i.e., a rapid change in time with respect to the genotypes [12,13]. Moreover, the application of insecticides has potential to maintain clones with different levels of resistance in population, and evolution of resistance may have the possibility of dramatic shifts in clonal frequencies. However, information regarding the genetic diversity of *A. gossypii* in Korea is currently unavailable. In Korea, *A. gossypii* is an important insect pest of pepper in greenhouses because greenhouses provide sustained warm temperature. Since greenhouses are a relatively closed environment, pest populations in greenhouses are generally more affected by chemical control and host plant changes than populations in fields. These practices may lead to reductions in pest population size and selection for resistant genotypes in populations, resulting in increased homozygosity within populations and differentiation between pest populations [14]. In addition, population dynamics may account for genetic variation of pest populations in greenhouses. Rochat et al. [15] revealed that a population of *A. gossypii* infesting cucurbit in greenhouses experiences extreme demographic fluctuations and strong founder effects through a small number of winged individuals immigrating from the exterior. Founder effects are common in aphid populations because of their high rate of population increase due to high fecundity and overlapping generations [16]. They have the potential to rapidly colonize surrounding plants, leading to very high-density infestations and inter-clonal competition [15]. Aphid populations in greenhouses may also experience local extinctions and serious bottlenecks due to plant resource exhaustion or insecticide treatments. Despite the importance of information on genetic variation, details on the genetic structure of *A. gossypii* in Korea are scarce.

Thus, the purpose of this study was to determine genetic diversity and geneflow of *A. gossypii* on greenhouse peppers (and a few field peppers) in Korea using microsatellite markers. We then discuss population genetic structure for this species based on the results, which may provide a practical framework for developing appropriate management strategies against *A. gossypii*.

## 2. Materials and Methods

### 2.1. Insect Samples

*A. gossypii* was collected in 18 populations from 2016, 14 populations in 2017, and five populations in 2018 in Korea (Table S1, Supplementary Materials). Most samples were collected from greenhouse peppers in summer season (late May to early August). However, GJ, CJ, YC, and GwJ were collected from field peppers in 2017 due to a change of cultivated crop or no occurrence of *A. gossypii*. All samples were placed in vials containing 95% ethanol and stored at −20 °C until DNA extraction.

### 2.2. Microsatellite Genotyping

DNA was extracted from individuals using a Qiagen Gentra Puregen Tissue Kit (Qiagen, MD, USA). A total of 1420 aphids were genotyped for eight microsatellite loci as described by Vanlerberghe-Masutti et al. [17]. The forward primer of each microsatellite locus was labeled with a fluorescent dye (FAM, NED, PET, VIC). Different dyes were chosen for loci having the same allele size to be analyzed simultaneously (Ago24-FAM, Ago53-VIC, Ago59-NED, Ago66-VIC, Ago69-NED, Ago84-PET, Ago89-PET, and Ago126-FAM). Polymerase chain reaction (PCR) was performed in two separate multiplex groups [12]. The first PCR, using primers specific to seven loci (Ago53, Ago59, Ago66, Ago69, Ago84, Ago89, and Ago126), was performed in a final volume of 10 µL containing 3.1 µL of distilled water, 1.0 µL of 10× PCR buffer, 1.0 µL of 10 mM dNTP mixture, 0.2 µL of each primer (final concentration, 10 pmol/µL), 0.1 µL of Taq polymerase (Takara Taq ™, Tokyo, Japan), and 2.0 µL of template DNA. Amplifications were performed in a thermocycler with the following parameters: initial denaturation at 95 °C for 15 min; 25 cycles of 30 s at 95 °C, 90 s at 56 °C, and 30 s at 72 °C; and a final extension for 30 min at 60 °C. The second PCR using primers specific to the eighth locus of Ago24 was performed in the same conditions except for distilled water volume (5.5 µL) with the following parameters: 5 min at 95 °C; 35 cycles of 30 s at 95 °C, 45 s at 62 °C, and 30 s at 72 °C; and a final elongation of 7 min at 72 °C. Then, 1 µL of each of these two PCR products was mixed with 8.5 µL of Hi-Di Formamide (Applied Biosystems, Foster City, USA) for denaturing and 0.5 µL of GeneScan™ 500 ROX™ Size Standard (Applied Biosystems, Foster City, USA). PCR products were separated and detected by capillary electrophoresis with an ABI 3730xl automatic sequencer (Applied Biosystems, Foster, USA) using the GENESCAN-500 [Rox] size standard. The genotype data were analyzed using GeneMapper 3.7 (Applied Biosystems, Foster City, USA).

### 2.3. Genetic Variation and Genetic Structure

Microsatellite genotype data of *A. gossypii* were analyzed with Micro-Checker [18] for the existence of null alleles, scoring errors, and large allele dropout. The confidence interval for Monte Carlo simulations of homozygote frequencies was set to 95%.

Basic parameters were analyzed to measure genetic diversity in *A. gossypii* population. An exact test for Hardy–Weinberg equilibrium (HWE) was conducted per locus and over all loci in each population using Genepop v. 4.2.1 [19]. Significance was tested using the Markov chain method (10,000 dememorizations, 100 batches, and 5000 iterations per batch). We used Poppr package [20] of R software version 3.5.0 to identify the multilocus genotypes [21]. Number of alleles (*N*_A_), observed heterozygosity (*H*_O_), expected heterozygosity (*H*_E_), and inbreeding coefficient (*F*_IS_) were calculated using GenAlEx version 6.5 [22]. Allelic richness (*A*_R_) was estimated using FSTAT version 2.9.3.2 [23]. *N*_A_, *H*_O_, *H*_E_, polymorphic information content (PIC), and measures of genetic diversity for each locus and averaged across loci were calculated with CERVUS software [24]. An *F*_ST_ value of zero implies a lack of divergence between populations, while an *F*_ST_ of one implies complete isolation of the population. Index of pairwise *F*_ST_ of Weir and Cockerham [25] between population and their associate 95% confidence intervals were estimated using FSTAT software. The ENA (excluding null alleles) method was also used to verify unbiased pairwise *F*_ST_ values (*F*_ST_^[ENA]^) using the FreeNA program [26].

Bottleneck events in *A. gossypii* populations were tested with BOTTLENECK program version 1.2 [27] using a two-phase model (TPM) and stepwise mutation model (SMM). We excluded the infinite alleles model (IAM) because IAM was not appropriate for microsatellites due to high microsatellite mutation rates and mutation processes that might retain memory of ancestral allelic states [28,29]. The SMM model can predict all mutations corresponding to increment or decrement of a single base-pair repeat. The TPM model observes the occurrence of an occasional multiple base-pair repeat [30]. It is suggested that TPM can closely simulate microsatellite mutation [31]. Thus, we used both TPM and SMM models. They are widely adopted for use with microsatellite markers [32,33]. Parameters chosen for TPM were as follows: variance = 30.00, probability = 70.00%, and estimations based on 10,000 iterations. Deviations from equilibrium were examined using Wilcoxon signed-rank test with significance level *p* < 0.05. The Wilcoxon signed-rank test is efficient and reliable when eight microsatellite loci are analyzed [31]. The genetic bottleneck test was reconfirmed through a mode shift indicator test based on a qualitative descriptor of allele frequency distribution.

Hierarchical analysis of molecular variance (AMOVA) was performed using GenAlEx that partitioned genetic variation among populations and individuals within populations. Significance of AMOVA analysis was estimated using 10,000 permutations. Isolation by distance (IBD) was analyzed by regressing pairwise population estimates of linearized *F*_ST_/ (1 − *F*_ST_) [34] on a natural log of the geographical distance between all pairs of sample location. Mantel’s test was implemented with 9999 permutation using ADEGENET package [35] of R software version 3.5.0 [21].

Population structure of *A. gossypii* was calculated by the Bayesian clustering procedure using STRUCTURE 2.3.3 [36] to explore different numbers of populations *K* to the population structure based on microsatellite data. Ten replicate runs with 600,000 MCMC (Markov chain Monte Carlo) iterations and a burn-in of 60,000 steps were performed for 1 ≤ *K* ≤ 10 (*K* = number of clusters) to verify the consistency of estimates and determine the most likely number of genetic clusters. The optimal value of *K* was determined using STRUCTURE HARVESTER [37] to compute Δ*K* [38]. Clustering pattern was applied to the CLUMPP program and visualized using DISTRUCT software version 1.1 [39]. Population structure was estimated using principal coordinate analysis (PCoA) applied in GenAlex, and then scatter diagram was plotted based on factor scores along the two PCoAs revealing the most variations. PCoA was based on the covariance of the genetic distance matrix, and analysis was implemented separately for each year. Discriminant analysis of principal component (DAPC) was carried out using ADEGENET package [35] of statistical package R software version 3.5.0 [21].

## 3. Results

In this study, a total of 57 alleles were verified across the eight microsatellite loci for 1420 *A. gossypii* individuals from 37 locations in Korea (18 populations in 2016, 14 populations in 2017, and five populations in 2018). Fisher’s exact tests showed that 291 of 1036 locus/population combinations deviated significantly from Hardy–Weinberg equilibrium (HWE). The number of alleles (*N*_A_), allelic richness (*A*_R_), observed heterozygosity (*H*_O_), expected heterozygosity (*H*_E_), and inbreeding coefficient (*F*_IS_) of populations are presented in Table 1. *N*_A_ ranged from 2.750 (JiJ_16) to 6.375 (JE_16), with an average of 4.091 across populations. *A*_R_ ranged from 2.707 (JiJ_16) to 5.949 (BS_16), with an average of 3.947. The lowest *H*_O_ was detected in JJ_17 (0.466), while the highest was detected in BS_16 (0.878). The lowest *H*_E_ was observed in GwJ_16 (0.354), while BS_16 showed the highest *H*_E_ (0.719). Inbreeding coefficient (*F*_IS_) ranged from −0.810 (JiJ_16) to 0.128 (JJ_17), with an average of −0.256 across populations. The presence of potential null alleles was indicated by a general excess of homozygotes for most allele size classes for no or one loci within at each population. The most polymorphic marker with the highest *N*_A_ per locus was 10 (Ago66), and the mean of *N*_A_ per locus was 7.125. Polymorphic information content (PIC) per locus ranged from 0.425 (Ago53) to 0.760 (Ago66). PIC values showed that all markers were informative (Table S2, Supplementary Materials).

Genetic structures of 37 geographic populations in 2016 (18 populations), 2017 (14 populations), and 2018 (five populations) were analyzed using pairwise comparisons of multilocus *F*_ST_ with or without ENA correction (*F*_ST_^[ENA]^) (Tables S3–S5, Supplementary Materials). Pairwise *F*_ST_ values between the populations in 2016 ranged from 0.0047 for GJ_16 and GS_16 populations (*F*_ST_^[ENA]^ = 0.0093; GJ_16 and GS_16 populations) to 0.4396 for YC_16 and GwJ_16 populations (*F*_ST_^[ENA]^ = 0.4287; YC_16 and GwJ_16 populations) (Table S3, Supplementary Materials). Pairwise *F*_ST_ values between populations in 2017 ranged from 0.0080 for AD_17 and GwJ_17 populations (*F*_ST_^[ENA]^ = 0.0109; AD_17 and GwJ_17 populations) to 0.3819 for CY_17 and CJu_17 populations (*F*_ST_^[ENA]^ = 0.3718; CY_17 and CJu_17 populations) (Table S4, Supplementary Materials). Pairwise *F*_ST_ values between populations in 2018 ranged from 0.0033 for CJu_18 and BS_18 populations (*F*_ST_^[ENA]^ = 0.0043; CJu_18 and BS_18 populations) to 0.2022 for CJu_18 and JJ_18 populations (*F*_ST_^[ENA]^ = 0.2008; CJu_18 and JJ_18 populations) (Table S5, Supplementary Materials). *F*_ST_ adjusted for null alleles and *F_ST_* assuming no null allele results were similar to each other. Overall *F*_ST_ value (uncorrected *F_ST_* = 0.1888, 95% confidence interval (CI): 0.1222–0.2532; ENA corrected *F*_ST_ = 0.1794, 95% confidence interval (CI): 0.1166–0.2411) indicated a high level of genetic differentiation among geographic populations.

Based on bottleneck analysis, significant heterozygote excess was shown in 10 of 37 populations under the two-phase model (TPM) and three of 37 populations using the stepwise mutation model (SMM). Accordingly, there was potential evidence for recent population reductions in a few populations. Most populations had a normal L-shaped distribution (except for YC_16, JiJ_16, and HS_18), indicating that *A. gossypii* expanded spatially without severe bottleneck in most regions of Korea. Population YC_16, JiJ_16, and HS_18 showed a significant bottleneck effect at *p* = 0.05 and a shifted shape (S) allele frequency distribution, providing evidence for recent population reduction in these populations (Table 2).

**Figure 1:**
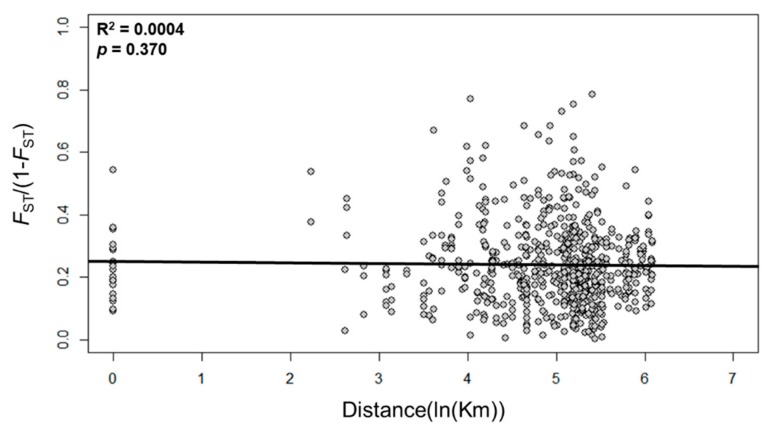
Geographical distance versus genetic distance (*F*_ST_/1 − *F*_ST_) for populations of *Aphis gossypii*, using pairwise *F*_ST_. Correlations and probabilities were estimated from a Mantel test with 9999 bootstrap repeats.

Analysis of molecular variance (AMOVA) for *A. gossypii* populations revealed a high variance component within individuals (85%), followed by among populations (15%) (Table 3). The component of variance among populations was significant (*F*_ST_). Based on the Mantel test for IBD, no significant correlation was found between genetic and geographical distances among populations (*r^2^* = 0.0004, *p* = 0.370), suggesting high gene flow among populations in Korea (Figure 1).

**Figure 2:**
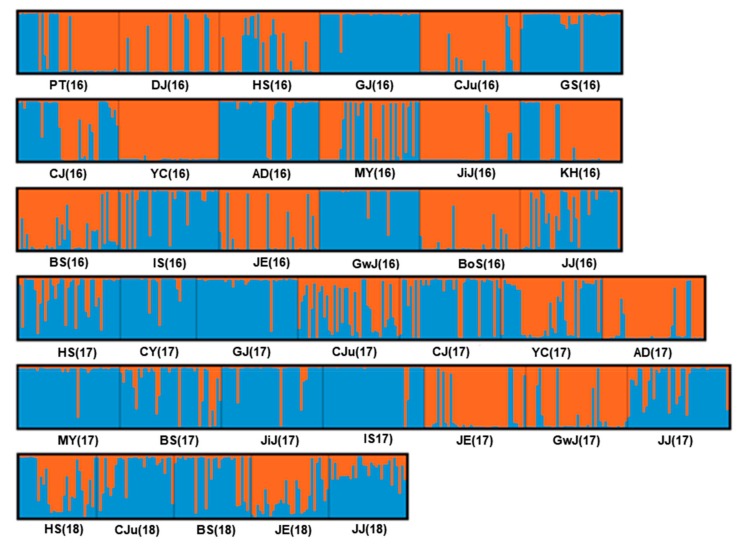
Structure profile under *K* = 2, permuted in CLUMPP, plotted with DISTRUCT on 37 *A. gossypii* populations, depicting classifications with the highest probability under the model that assumes independent allele frequencies and inbreeding coefficients among assumed clusters. Each individual is represented by a vertical bar, often partitioned into colored segments with the length of each segment representing the proportion of the individual’s genome from *K* = 2 ancestral populations. On the bottom of the plot, the name of population localities is indicated and the year of sampling is shown in parentheses.

**Figure 3:**
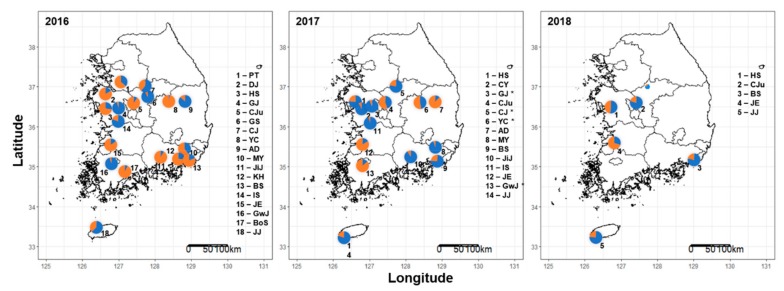
All populations partitioned in two clusters (*K* = 2) and the pie graphs revealing the results from a Bayesian cluster analysis of multilocus genotypes in 2016, 2017, and 2018. The population identifiers (IDs) are indicated in pie graphs (* = samples collected from field pepper).

Results of Bayesian analysis of population genetic structure indicated that the best dataset partitioning involved two genetic clusters since the value of Δ*K* (Evanno method) occurred at *K* = 2 with a maximum value of 1251.3 (Figure S1, Supplementary Materials). Using partition *K* = 2, graphics were drawn with DISTRUCT to visualize the clustering pattern of individuals and populations (Figure 2). In addition, results of Bayesian cluster analysis of multilocus microsatellite genotypes in 2016, 2017, and 2018 are displayed as pie graphs (Figure 3). Opposite genetic clusters appeared between 2016 and 2017, mostly in northwest (HS, CJu) and southeast (MY, BS, JiJ) parts of Korea, although samples were collected from the same pepper greenhouse. HS population showed an opposite pie graph compared to 2016 and 2017. However, the cluster was divided in half in 2018. JE and BS populations showed similar pie graphs in 2017, while CJu showed the process of changing to opposite cluster.

**Figure 4:**
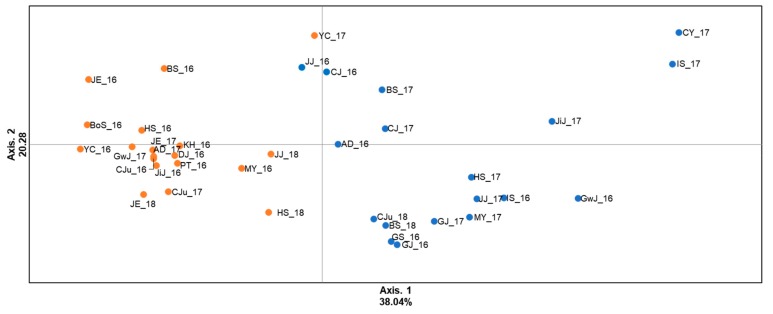
Scatter diagram of factor scores from a principal coordinate analysis of genotype data for eight microsatellite loci in samples of *A. gossypii* collected from 37 locations in Korea (2016, 2017, and 2018). The percentage of total variation attributed to each axis is indicated.

Principal coordinate analysis (PCoA) showed that the pattern of genetic structure was similar to the genetic cluster analysis result (two distinct groups) (Figure 4). Total variance explained by the first and second axes in 2016, 2017, and 2018 was 56% (30.39% for axis 1 and 25.11% for axis 2). Discriminant analysis of principal component (DAPC) results were highly similar to PCoA based on the scatter plot of the population. However, most populations were at the gravity center because genetic variation between populations was not large enough to be divided into two distinct groups. (Figure S2, Supplementary Materials).

## 4. Discussion

This study is the first attempt to understand the pattern of genetic variability in *A. gossypii* in Korea. In the present study, we analyzed genetic diversity within and between *A. gossypii* populations collected from pepper plants (mostly in greenhouses). Genetic variability found in *A. gossypii* through the use of molecular markers revealed that its clonal diversity is structured by its host plants [17]. In the present study, *A. gossypii* had low to moderate genetic diversity based on microsatellite data (Table 1), in which *H*_E_ varied from 0.354 (GwJ_16) to 0.719 (BS_16). Most *H*_E_ values were lower than the average (average = 0.524). A similar level of genetic variation was shown in other aphid species (*Sitobion avenae*), for which H*_E_* ranged from 0.409 to 0.873 on the basis of eight microsatellite loci [40]. Within a greenhouse population of *A. gossypii*, clonal diversity declined significantly as spring/summer season progressed [16]. Similarly, in *S. avenae*, genetic variation significantly decreased from spring to summer [41]. Brévault et al. [42] revealed that *A. gossypii* populations, collected from cotton crops, vegetable crops, and weeds in northern Cameroon, show very low genetic diversity (only 11 multilocus genotypes identified). The final predominance of the clone may occur through a combination of genetic drift related to population foundation, demographic explosion, and/or clonal competition. The final fittest founder genotype will become dominant during the period of rapid population growth, while recessive genotypes will decrease in frequency or may extinct completely. Moreover, *Eriosoma lanigerum* and *Myzus persicae* show low levels of genetic diversity due to adaptation of aphids to heavy selection pressures, including distribution of host plants and the use of insecticides [43,44]. Cyclical parthenogenesis may explain the low level of genetic variability detected in aphids compared to other insects. It could be an important factor that leads to local or temporal genetic differentiation of populations [45]. In our study, most *A. gossypii* samples were collected from greenhouse peppers in the summer, during which most *A. gossypii* were under cyclical parthenogenesis. Chemical control was common in all greenhouses. This apparently led to heavy selective pressure for *A. gossypii*. These points may explain such low levels of genetic variability of *A. gossypii* observed in Korea. Moreover, *A. gossypii* developed resistance to insecticides such as carbamates and organophosphates. Its exponential growth due to parthenogenesis favors rapid selection for insecticide resistance traits [46,47]. Therefore, it would be important to understand the genetic structure and level of gene flow of *A. gossypii* populations, to estimate insecticide resistance over temporal and spatial scales and investigate distribution of resistance genes among *A. gossypii* populations because resistance genes are related to genetic factors.

The moderate flight and dispersal ability of aphids might allow short-distance movement. Usually, the IBD test is widely used to examine spatial patterns of gene flow and genetic relatedness between populations [48]. Results of IBD analysis revealed that geographic distance had no effect on *A. gossypii* population structure in Korea (Figure 1). In the present study, the lack of a significant IBD pattern may indicate unrestricted gene flow among *A. gossypii* populations in Korea. Genetic structure analysis based on STRUCTURE and PCoA revealed two genetic clusters in *A. gossypii* populations in Korea (Figure 2 and Figure 3). Interestingly, different genetic clusters were found between 2016 and 2017, mostly in northwest (HS, CJu, IS) and southeast (MY, BS, JiJ) parts of Korea, although samples were collected from the same greenhouses. Interestingly, Jeju (JJ) population showed similar genetic clusters during three years. Such similar genetic structures might be due to limited immigration as growers raise pepper seedlings by themselves and Jeju is an island. Within a greenhouse, clonal composition does not persist over time. These changes might result from local extinction (due to insecticide, crop change, cold winter temperature, etc.), followed by recolonization by winged immigrants coming from neighboring greenhouses, from refuges, or from raising of seedlings. In addition, different clusters observed over the years can be attributed to a founder effect. A greenhouse is a comparatively enclosed space. Once it is founded, migration to the outside would be limited and vice versa. A few new immigrants that succeed in dispersing into a greenhouse after insecticide treatment for control might have a significant effect on the genetic structure of *A. gossypii*. Moreover, the population structure of A. *gossypii* might change dramatically due to bottlenecks and rapid gene flow, because several locations (HS, CJu, BS, JiJ) showed a significant *p*-value in the TPM model. Moreover, a change in genetic cluster in a period of one year might be caused by different fitness between two genetic clusters of *A. gossypii* in Korea. Two genetic clusters of *A. gossypii* might have coexisted in the same regions, and one genetic cluster could be a dominant genotype if there is a fitness difference between them.

## 5. Conclusions

Genetic diversity was revealed among *A. gossypii* populations in Korea based on eight microsatellite loci. *A. gossypii* populations in Korea appeared to be classified into two genetic clusters. However, its genetic structure rapidly changed into opposite clusters in several regions, although samples were collected from the same locations. Based on the results of bottleneck analysis (TPM, SMM model), most *A. gossypii* populations experienced a recent spatial expansion without any severe bottleneck in most regions of Korea. However, several locations (HS, CJu, BS, JiJ) were shown to be significant in TPM, which may affect the rapid turnover in genetic structure. These results provide important information for understanding its feasible local adaptation and dispersal patterns. *A. gossypii* populations in pepper-growing (field and greenhouse) areas in Korea showed low genetic diversity and high gene flow due to cyclical parthenogenesis and heavy insecticidal selection pressure. Thus, future work should elucidate any phenotypic fitness difference between the two genetic clusters of *A. gossypii* in Korea. Also, further studies should focus on the relationship between the genetic structure of *A. gossypii* populations in various crop-producing areas and the extent of insecticide selection pressure.

## Figures and Tables

**Table 1:** Genetic variation estimates of geographic population of *Aphis gossypii*. Number of alleles (*N*_A_), allelic richness (*A*_R_), observed heterozygosity (*H*_O_), expected heterozygosity (*H*_E_), inbreeding coefficient (*F*_IS_), probability (*p*-value) of being in Hardy–Weinberg equilibrium (HWE), and loci showing potential null alleles. ID—identifier.

Sampling Name	Population ID	Sample Size	N*_A_*	*A*_R_	*H*_O_	*H*_E_	*p*-Value	*F*_IS_^1^	Loci with Null Alleles
PT	PT_16	40	4.250	4.124	0.759	0.575	0.00002	−0.257 ***	Ago53
DJ	DJ_16	40	3.250	3.172	0.744	0.502	0.00002	−0.438 ***	No
HS	HS_16	40	5.250	5.080	0.741	0.623	0.00002	−0.172 ***	Ago69
GJ	GJ_16	40	3.000	2.735	0.616	0.404	0.00002	−0.322 ***	Ago66, Ago126
CJu	CJu_16	40	3.500	3.361	0.828	0.510	0.00002	−0.548 ***	No
GS	GS_16	40	3.250	3.014	0.594	0.417	0.00002	−0.297 ***	Ago66
CJ	CJ_16	40	5.500	5.266	0.851	0.709	0.00002	−0.214 ***	No
YC	YC_16	40	2.875	2.803	0.716	0.442	0.00002	−0.554 ***	No
AD	AD_16	40	5.625	5.334	0.750	0.623	0.00002	−0.241 ***	Ago53
MY	MY_16	40	4.625	4.429	0.694	0.570	0.00002	−0.178 ***	Ago53, Ago69
JiJ	JiJ_16	40	2.750	2.707	0.850	0.472	0.00002	−0.810 ***	No
KH	KH_16	40	4.625	4.580	0.766	0.635	0.00002	−0.168 ***	Ago53
BS	BS_16	40	6.125	5.949	0.878	0.719	0.00002	−0.228 ***	No
IS	IS_16	40	5.000	4.736	0.541	0.516	0.00002	−0.025 ***	Ago53, Ago66, Ago126
JE	JE_16	40	6.375	5.919	0.859	0.656	0.00002	−0.315 ***	No
GwJ	GwJ_16	40	3.375	3.161	0.544	0.354	0.00002	−0.373 ***	Ago53
Bos	BoS_16	40	4.500	4.299	0.850	0.561	0.00002	−0.502 ***	No
JJ	JJ_16	40	5.625	5.363	0.687	0.632	0.00002	−0.078 ***	Ago59
HS	HS_17	40	3.375	3.271	0.494	0.422	0.00002	−0.110 ***	Ago53, Ago59, Ago66
CY	CY_17	30	3.625	3.625	0.513	0.438	0.00002	−0.205 ***	Ago53
GJ	GJ_17 *	40	3.000	2.883	0.559	0.433	0.00002	-0.166***	Ago53, Ago59, Ago66
CJu	CJu_17	40	3.000	2.904	0.631	0.469	0.00002	−0.277 ***	Ago59, Ago69
CJ	CJ_17 *	40	4.875	4.566	0.719	0.574	0.00002	−0.217 ***	Ago53
YC	YC_17 *	40	3.750	3.639	0.741	0.563	0.00002	−0.289 ***	No
AD	AD_17	40	3.250	3.203	0.725	0.511	0.00002	−0.332 ***	Ago53, Ago69
MY	MY_17	40	3.750	3.611	0.547	0.428	0.00002	−0.048 ***	Ago53, Ago59, Ago69, Ago126
BS	BS_17	40	5.375	5.131	0.691	0.602	0.00002	−0.155 ***	Ago53, Ago59, Ago126
JiJ	JiJ_17	40	3.625	3.461	0.656	0.549	0.00002	−0.235 ***	Ago53, Ago66
IS	IS_17	40	3.875	3.641	0.616	0.473	0.00002	−0.301 ***	Ago53
JE	JE_17	40	3.875	3.708	0.763	0.527	0.00002	−0.346 ***	No
GwJ	GwJ_17 *	40	4.375	4.131	0.734	0.527	0.00002	−0.280 ***	Ago53
JJ	JJ_17	40	4.875	4.620	0.466	0.477	0.00002	0.128 ***	Ago24, Ago53, Ago59
HS	HS_18	30	2.875	2.875	0.608	0.513	0.00002	−0.138 ***	Ago53, Ago59, Ago69
CJu	CJu_18	30	3.250	3.250	0.579	0.501	0.00002	−0.164 ***	Ago53, Ago59, Ago69
BS	BS_18	30	3.500	3.500	0.550	0.485	0.00002	−0.107 ***	Ago53, Ago59, Ago69
JE	JE_18	30	3.250	3.250	0.596	0.443	0.00002	−0.322 ***	Ago59, Ago69
JJ	JJ_18	30	4.375	4.375	0.700	0.542	0.00002	−0.171 ***	Ago53, Ago69

* *A. gossypii* was collected from field pepper; HW test: Hardy–Weinberg exact test [19] with Bonferroni correction (*p* = 0.000017). ^1^ Significance *F*_IS_ value was obtained after a 1000-permutation test (** *p* < 0.05; *** *p* < 0.01).

**Table 2:** Wilcoxon signed-rank test for mutation-drift equilibrium estimated based on eight microsatellite loci.

Population ID	TPM	SMM	Mode Shift	Population ID	TPM	SMM	Mode Shift
PT_16	0.191	0.680	L	CY_17	0.281	0.578	L
DJ_16	0.037 **^,2^	0.156	L	GJ_17 *	0.191	0.422	L
HS_16	0.371	0.629	L	CJu_17	0.020 **	0.273	L
GJ_16	0.422	0.473	L	CJ_17 *	0.371	0.680	L
CJu_16	0.098	0.273	L	YC_17 *	0.004 **	0.006 **	L
GS_16	0.289	0.813	L	AD_17	0.125	0.371	L
CJ_16	0.002 **	0.037 **	L	MY_17	0.727	0.844	L
YC_16	0.027 **	0.188	S	BS_17	0.527	0.809	L
AD_16	0.727	0.994	L	JiJ_17	0.027 **	0.098	L
MY_16	0.422	0.770	L	IS_17	0.371	0.809	L
JiJ_16	0.004 **	0.020 **	S	JE_17	0.098	0.191	L
KH_16	0.027 **	0.230	L	GwJ_17 *	0.473	0.875	L
BS_16	0.010 **	0.473	L	JJ_17	0.973	0.994	L
IS_16	0.809	0.990	L	HS_18	0.014 **	0.098	S
JE_16	0.629	0.963	L	CJu_18	0.098	0.273	L
GwJ_16	0.711	0.813	L	BS_18	0.191	0.527	L
BoS_16	0.422	0.727	L	JE_18	0.289	0.594	L
JJ_16	0.473	0.963	L	JJ_18	0.320	0.809	L
HS_17	0.469	0.469	L				

^2^*p* is test for heterozygosity excess, ** *p* < 0.05; TPM: two-phase model; SMM: stepwise mutation model; L: normal L-shaped distribution S: shifted mode.

**Table 3:** Analysis of molecular variance (AMOVA) analysis on eight microsatellites in different populations of *A. gossypii* in Korea (*** *p* < 0.01). df—degrees of freedom

Source of Variation	df	Sum of Squares	Mean Sum of Squares	Estimated Variance	% of Variation	*F*-Statistics
Among populations	36	1434.821	39.856	0.500	15%	*F*_ST_ = 0.190 ***
Among individuals within populations	1383	2099.471	1.518	0.000	0%	*F*_IS_ = −0.286
Within individuals	1420	3879.000	2.732	2.732	85%	*F*_IT_ = −0.041
Total	2839	7413.292		3.231	100%	

## Supplementary Materials

The following are available online at https://www.mdpi.com/2075-4450/10/10/319/s1: Figure S1: Bayesian inference to identify suitable cluster (K) and cluster proportion using STRUCUTRE for *Aphis gossypii* in Korea. Delta K are analyzed against number of genetic clusters (K); Figure S2: Scatter plot of DAPC analysis of the nine populations using “adegent” in R package (Table 2 indicates the population ID). Dots: individuals, ellipses: populations; Table S1: Sampling information of *A. gossypii* collected in Korea (2016, 2017, and 2018); Table S2: Microsatellite loci, number of alleles observed at each locus (NA), observed (HO) and expected (HE) heterozygosity at each locus, and mean PIC (polymorphic information content) per locus in *A. gossypii* population; Table S3: Pairwise FST[ENA] values (lower-left matrix), and pairwise FST values and significance (upper-right matrix) based on eight microsatellite loci between the populations *of A. gossypii* in Korea (2016); Table S4: Pairwise FST[ENA] values (lower-left matrix), and pairwise FST values and significance (upper-right matrix) based on eight microsatellite loci between the populations of *A. gossypii* in Korea (2017); Table S5: Pairwise FST[ENA] values (lower-left matrix), and pairwise FST values and significance (upper-right matrix) based on eight microsatellite loci between the populations of *A. gossypii* in Korea (2018).
