# Supplementary material for: Population Genetic Structure of Aphis gossypii Glover (Hemiptera: Aphididae) in Korea
Source: Insects. 2019 Sep 26;10(10):319. doi: 10.3390/insects10100319 (PMC6835795; doi:10.3390/insects10100319)
Supplement: Supplementary file 1 [file insects-10-00319-s001.zip › Supplementary Fig.1.pdf]

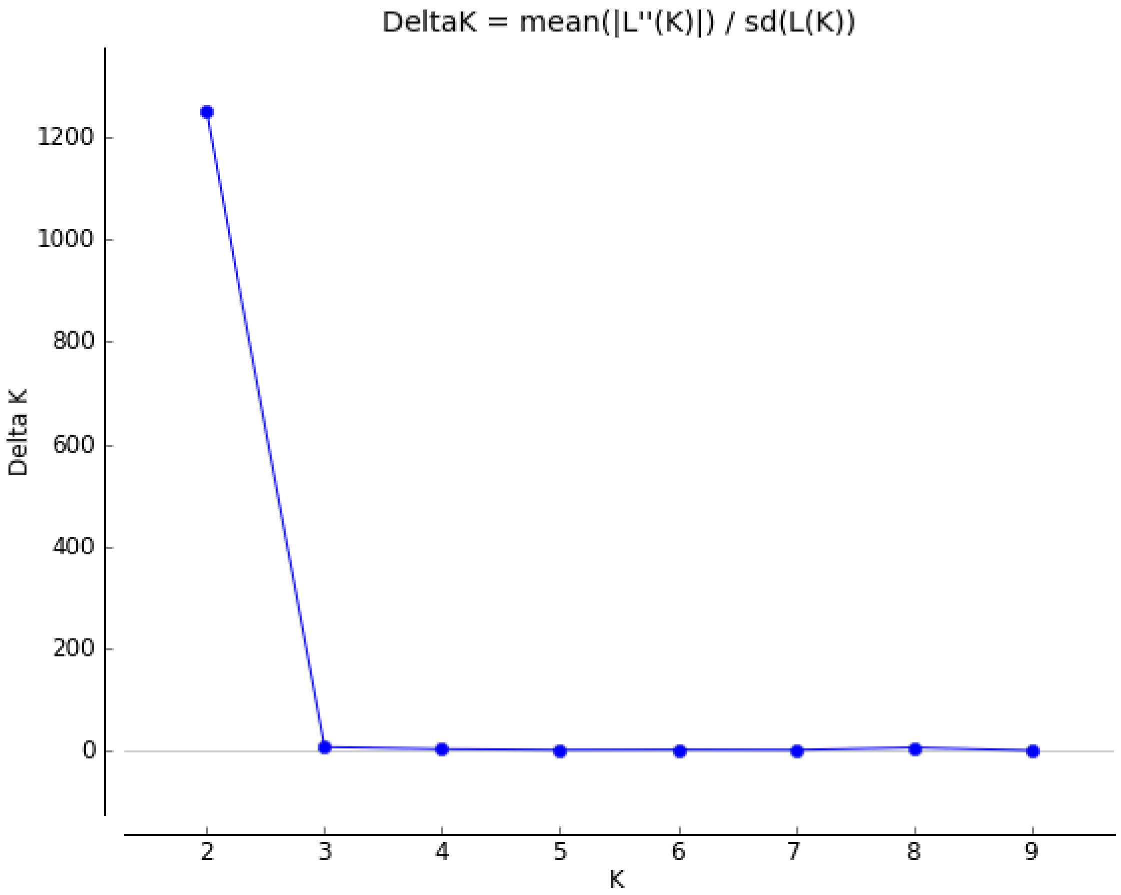

Supplementary Figure 1. Bayesian inference to identify suitable cluster (*K*) and cluster proportion using STRUCUTRE for *Aphis gossypii* in Korea. Delta *K* are analyzed against number of genetic clusters (*K*).
