# Supplementary material for: Population Genetic Structure of Aphis gossypii Glover (Hemiptera: Aphididae) in Korea
Source: Insects. 2019 Sep 26;10(10):319. doi: 10.3390/insects10100319 (PMC6835795; doi:10.3390/insects10100319)
Supplement: Supplementary file 1 [file insects-10-00319-s001.zip › Supplementary Fig.2.pdf]

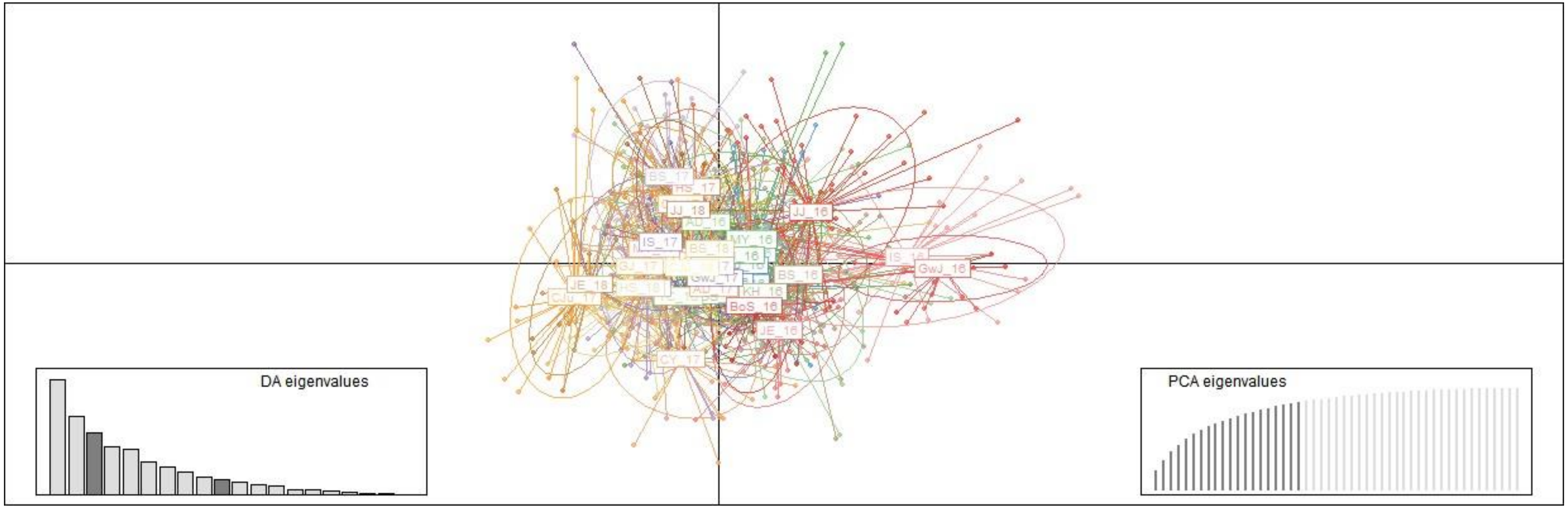

Supplementary Figure S2. Scatter plot of DAPC analysis of the nine populations using "adegent" in R package (table 2 indicates the population ID). Dots: individuals, ellipses: populations.
