# Supplementary material for: Population Genetic Structure of Aphis gossypii Glover (Hemiptera: Aphididae) in Korea
Source: Insects. 2019 Sep 26;10(10):319. doi: 10.3390/insects10100319 (PMC6835795; doi:10.3390/insects10100319)
Supplement: Supplementary file 1 [file insects-10-00319-s001.zip › Supplementary Table 1.docx]

Supplementary Table 1. Sampling information of *A. gossypii* collected in Korea (2016, 2017, and 2018).

| Country | Sampling name | Sampling Site | Sampling Date | Coordinates |
| --- | --- | --- | --- | --- |
| Korea | JiJ | Jinjushi, Kyunsagnam-do | 2016-05-25  2017-06-07 | N35°13'51.0",E128°08'12.0" |
|  | BS | Busan Metropolitan city, Kyunsagnam-do | 2016-05-26  2017-06-08  2018-07-11 | N35°10'18.0",E128°54'55.0" |
|  | KH | Kimhaeshi, Kyungsangnam-do | 2016-05-26 | N35°20'14.0",E128°46'38.0" |
|  | MY | Milyangshi, Kyunsagnam-do | 2016-05-27  2017-06-08 | N35°26'56.0",E128°48'29.0" |
|  | JE | Jeongeupshi, Jeonlabuk-do | 2016-06-01  2017-06-20  2018-07-11 | N35°33'42.8",E126°49'03.5" |
|  | IS | Iksanshi, Jeonlabuk-do | 2016-06-03  2017-06-20 | N36°08'21.0",E126°58'59.0" |
|  | BoS | Boseonggeun, Jeonlanam-do | 2016-06-02 | N34°53'23.0",E127°09'44.0" |
|  | GwJ | Gwangju Metropolitan city, Jeonlanam-do | 2016-06-02  2017-06-02^*^ | N35°03'45.0",E126°49'20.0"  N35°03'45.7",E126°49'49.4" |
|  | AD | Andongshi, Kyungsangbuk-do | 2016-06-09  2017-06-20 | N36°37'57.0",E128°46'52.0" |
|  | YC | Yecheonguen, Kyungsanbuk-do | 2016-06-09  2017-06-27^*^ | N36°37'15.0",E128°22'39.0"  N36°37'14.9",E128°22'39.2" |
|  | DJ | Dangjinshi, Chungcheongnam-do | 2016-06-29 | N36°49'10.0",E126°38'29.0" |
|  | HS | Hongseongguen, Chungcheongnam-do | 2016-06-29  2017-06-26  2018-07-13 | N36°30'49.0",E126°42'35.0" |
|  | CY | Cheongyangguen, Chungcheongnam-do | 2017-06-21 | N36°28'59.0",E126°51'02.0" |
|  | GJ | Gongjushi, Chungcheongnam-do | 2016-06-30  2017-06-21^*^ | N36°29'45.0",E126°56'32.0"  N36°29'45.0",E126°56'32.1" |
|  | CJu | Cheongju, Chungcheongbuk-do | 2016-06-30  2017-06-26  2018-07-12 | N36°35'34.0",E127°25'41.0" |
|  | GS | Goesangeun, Chungchceongbuk-do | 2016-07-01 | N36°51'28.0",E127°45'21.0" |
|  | CJ | Chungju, Chungcheongbuk-do | 2016-07-01  2017-06-19^*^ | N36°59'58.0",E127°43'32.0"  N36°59'58.0",E127°43'32.1” |
|  | PT | Pyeongtaekshi, Gyeonggi-do | 2016-08-05 | N37°07'23.0",E127°03'55.0" |
|  | JJ | Jejushi, Jejudo  Seogwiposhi, Jejudo | 2016-04-25  2017-06-26  2018-10-10 | N33°28'59.8",E126°23'05.7"  N33°16'03.0",E126°15'47.6"  N33°16'03.0",E126°15'47.6" |

* *A. gossypii* was collected from field pepper
