# Supplementary material for: Population Genetic Structure of Aphis gossypii Glover (Hemiptera: Aphididae) in Korea
Source: Insects. 2019 Sep 26;10(10):319. doi: 10.3390/insects10100319 (PMC6835795; doi:10.3390/insects10100319)
Supplement: Supplementary file 1 [file insects-10-00319-s001.zip › Supplementary Table 2.docx]

Supplementary Table 2. Microsatellite loci, number of alleles observed at each locus (*N*_A_), observed (*H*_O_) and expected (*H*_E_) heterozygosity at each locus, and mean PIC (polymorphic information content) per locus in *A. gossypii* population.

| Locus | *N*_A_ | *H*_O_ | *H*_E_ | PIC |
| --- | --- | --- | --- | --- |
| Ago24 | 6 | 0.848 | 0.618 | 0.544 |
| Ago53 | 6 | 0.182 | 0.442 | 0.425 |
| Ago59 | 8 | 0.665 | 0.790 | 0.758 |
| Ago66 | 10 | 0.739 | 0.791 | 0.760 |
| Ago69 | 9 | 0.588 | 0.775 | 0.743 |
| Ago84 | 5 | 0.982 | 0.651 | 0.585 |
| Ago89 | 5 | 0.992 | 0.581 | 0.494 |
| Ago126 | 8 | 0.472 | 0.559 | 0.514 |
|  | 7.125 | 0.684 | 0.651 | 0.603 |
