# Supplementary material for: Population Genetic Structure of Aphis gossypii Glover (Hemiptera: Aphididae) in Korea
Source: Insects. 2019 Sep 26;10(10):319. doi: 10.3390/insects10100319 (PMC6835795; doi:10.3390/insects10100319)
Supplement: Supplementary file 1 [file insects-10-00319-s001.zip › Supplementary Table 3.docx]

Supplementary Table 3. Pairwise *F*_ST_ ^[ENA]^ values (lower-left matrix), and pairwise *F*_ST_ values and significance (upper-right matrix) based on 8 microsatellite loci between the populations of *A. gossypii* in Korea (2016).

|  | PT_16 | DJ_16 | HS_16 | GJ_16 | CJu_16 | GS_16 | CJ_16 | YC_16 | AD_16 | MY_16 | JiJ_16 | KH_16 | BS_16 | IS_16 | JE_16 | GwJ_16 | BoS_16 | JJ_16 |
| --- | --- | --- | --- | --- | --- | --- | --- | --- | --- | --- | --- | --- | --- | --- | --- | --- | --- | --- |
| PT_16 | - | 0.1376^*^ | 0.0514^*^ | 0.1766^*^ | 0.1167^*^ | 0.1549^*^ | 0.1091^*^ | 0.0783^*^ | 0.1030^*^ | 0.1007^*^ | 0.1503^*^ | 0.0930^*^ | 0.1329^*^ | 0.2228^*^ | 0.1083^*^ | 0.3425^*^ | 0.1319^*^ | 0.1387^*^ |
| DJ_16 | 0.1397 | - | 0.0721^*^ | 0.2321^*^ | 0.0629^*^ | 0.2275^*^ | 0.1396^*^ | 0.1313^*^ | 0.2376^*^ | 0.0368^*^ | 0.0144^NS^ | 0.0512^*^ | 0.1592^*^ | 0.2366^*^ | 0.1253^*^ | 0.3632^*^ | 0.1352^*^ | 0.1731^*^ |
| HS_16 | 0.0500 | 0.0713 | - | 0.1807^*^ | 0.0732^*^ | 0.1686^*^ | 0.0808^*^ | 0.0440^*^ | 0.1466^*^ | 0.0503^*^ | 0.0938^*^ | 0.0320^*^ | 0.0734^*^ | 0.1860^*^ | 0.0470^*^ | 0.3102^*^ | 0.0643^*^ | 0.1310^*^ |
| GJ_16 | 0.1590 | 0.2196 | 0.1565 | - | 0.2472^*^ | **0.0047^NS^** | 0.1967^*^ | 0.2906^*^ | 0.1722^*^ | 0.1497^*^ | 0.2652^*^ | 0.1622^*^ | 0.2380^*^ | 0.1331^*^ | 0.2518^*^ | 0.2433^*^ | 0.2805^*^ | 0.2445^*^ |
| CJu_16 | 0.1189 | 0.0628 | 0.0699 | 0.2319 | - | 0.2321^*^ | 0.1414^*^ | 0.1206^*^ | 0.2333^*^ | 0.0761^*^ | 0.0691^*^ | 0.0877^*^ | 0.1445^*^ | 0.2592^*^ | 0.1451^*^ | 0.3936^*^ | 0.1415^*^ | 0.1703^*^ |
| GS_16 | 0.1508 | 0.2204 | 0.1531 | **0.0093** | 0.2265 | - | 0.1896^*^ | 0.2684^*^ | 0.1734^*^ | 0.1493^*^ | 0.2612^*^ | 0.1551^*^ | 0.2274^*^ | 0.1449^*^ | 0.2369^*^ | 0.2645^*^ | 0.2595^*^ | 0.2306^*^ |
| CJ_16 | 0.1027 | 0.1417 | 0.0749 | 0.1787 | 0.1434 | 0.1772 | - | 0.1731^*^ | 0.1197^*^ | 0.0825^*^ | 0.1615^*^ | 0.0722^*^ | 0.0870^*^ | 0.1496^*^ | 0.0916^*^ | 0.2457^*^ | 0.1387^*^ | 0.0941^*^ |
| YC_16 | 0.0816 | 0.1328 | 0.0516 | 0.2744 | 0.1209 | 0.2630 | 0.1750 | - | 0.2503^*^ | 0.1232^*^ | 0.1474^*^ | 0.1077^*^ | 0.1799^*^ | 0.3127^*^ | 0.1167^*^ | **0.4396^*^** | 0.1155^*^ | 0.2097^*^ |
| AD_16 | 0.0984 | 0.2396 | 0.1414 | 0.1549 | 0.2361 | 0.1677 | 0.1184 | 0.2552 | - | 0.1757^*^ | 0.2485^*^ | 0.1570^*^ | 0.1489^*^ | 0.1863^*^ | 0.1786^*^ | 0.2736^*^ | 0.2411^*^ | 0.1818^*^ |
| MY_16 | 0.0997 | 0.0363 | 0.0453 | 0.1378 | 0.0749 | 0.1417 | 0.0757 | 0.1303 | 0.1688 | - | 0.0663^*^ | 0.0261^NS^ | 0.1237^*^ | 0.1359^*^ | 0.0997^*^ | 0.2606^*^ | 0.1074^*^ | 0.1239^*^ |
| JiJ_16 | 0.1525 | 0.0139 | 0.0921 | 0.2507 | 0.0692 | 0.2555 | 0.1632 | 0.1473 | 0.2515 | 0.0660 | - | 0.0791^*^ | 0.1819^*^ | 0.2679^*^ | 0.1591^*^ | 0.3960^*^ | 0.1686^*^ | 0.1953^*^ |
| KH_16 | 0.0927 | 0.0556 | 0.0292 | 0.1553 | 0.0919 | 0.1507 | 0.0678 | 0.1126 | 0.1546 | 0.0255 | 0.0843 | - | 0.0813^*^ | 0.1539^*^ | 0.0389^*^ | 0.2643^*^ | 0.0665^*^ | 0.1306^*^ |
| BS_16 | 0.1280 | 0.1598 | 0.0683 | 0.2148 | 0.1459 | 0.2119 | 0.0874 | 0.1804 | 0.1484 | 0.1147 | 0.1832 | 0.0776 | - | 0.2001^*^ | 0.0476^*^ | 0.3241^*^ | 0.0970^*^ | 0.1374^*^ |
| IS_16 | 0.2066 | 0.2377 | 0.1740 | 0.1330 | 0.2583 | 0.1375 | 0.1364 | 0.3116 | 0.1724 | 0.1265 | 0.2679 | 0.1459 | 0.1902 | - | 0.2280^*^ | 0.0526^NS^ | 0.2650^*^ | 0.2238^*^ |
| JE_16 | 0.1052 | 0.1263 | 0.0474 | 0.2374 | 0.1464 | 0.2281 | 0.0936 | 0.1174 | 0.1815 | 0.0955 | 0.1602 | 0.0369 | 0.0484 | 0.2234 | - | 0.3399^*^ | 0.0488^*^ | 0.1471^*^ |
| GwJ_16 | 0.3183 | 0.3527 | 0.2939 | 0.2337 | 0.3836 | 0.2360 | 0.2319 | **0.4287** | 0.2609 | 0.2412 | 0.3860 | 0.2453 | 0.3124 | 0.0573 | 0.3300 | - | 0.4014^*^ | 0.3462^*^ |
| BoS_16 | 0.1312 | 0.1359 | 0.0653 | 0.2662 | 0.1421 | 0.2534 | 0.1407 | 0.1157 | 0.2443 | 0.1051 | 0.1690 | 0.0679 | 0.0985 | 0.2619 | 0.0491 | 0.3912 | - | 0.1643^*^ |
| JJ_16 | 0.1330 | 0.1753 | 0.1227 | 0.2272 | 0.1713 | 0.2236 | 0.0883 | 0.2116 | 0.1748 | 0.1171 | 0.1966 | 0.1262 | 0.1319 | 0.2035 | 0.1442 | 0.3234 | 0.1640 | - |

*: *P*<0.05 (significant value); NS: not significant.
