# Supplementary material for: Population Genetic Structure of Aphis gossypii Glover (Hemiptera: Aphididae) in Korea
Source: Insects. 2019 Sep 26;10(10):319. doi: 10.3390/insects10100319 (PMC6835795; doi:10.3390/insects10100319)
Supplement: Supplementary file 1 [file insects-10-00319-s001.zip › Supplementary Table 4.docx]

Supplementary Table 4. Pairwise *F*_ST_ ^[ENA]^ values (lower-left matrix), and pairwise *F*_ST_ values and significance (upper-right matrix) based on 8 microsatellite loci between the populations of *A. gossypii* in Korea (2017).

|  | HS_17 | CY_17 | GJ_17 | CJu_17 | CJ_17 | YC_17 | AD_17 | MY_17 | BS_17 | JiJ_17 | IS_17 | JE_17 | GwJ_17 | JJ_17 |
| --- | --- | --- | --- | --- | --- | --- | --- | --- | --- | --- | --- | --- | --- | --- |
| HS_17 | - | 0.2496^*^ | 0.1068^*^ | 0.2702^*^ | 0.1000^*^ | 0.249^*^ | 0.2071^*^ | 0.1002^*^ | 0.1207^*^ | 0.1395^*^ | 0.2008^*^ | 0.2104^*^ | 0.2204^*^ | 0.1060^*^ |
| CY_17 | 0.2413 | - | 0.2738^*^ | **0.3819**^*^ | 0.1927^*^ | 0.2954^*^ | 0.3448^*^ | 0.2720^*^ | 0.1993^*^ | 0.1492^*^ | 0.2008^*^ | 0.3368^*^ | 0.3471^*^ | 0.2444^*^ |
| GJ_17 | 0.0924 | 0.2625 | - | 0.1865^*^ | 0.1045^*^ | 0.2857^*^ | 0.2139^*^ | 0.0229^NS^ | 0.1467^*^ | 0.1178^*^ | 0.2505^*^ | 0.2122^*^ | 0.2259^*^ | 0.0824^*^ |
| CJu_17 | 0.2536 | **0.3718** | 0.1710 | - | 0.1885^*^ | 0.2664^*^ | 0.2037^*^ | 0.2257^*^ | 0.1674^*^ | 0.2513^*^ | 0.3667^*^ | 0.1946^*^ | 0.2025^*^ | 0.2150^*^ |
| CJ_17 | 0.0967 | 0.1944 | 0.0937 | 0.1702 | - | 0.2004^*^ | 0.1558^*^ | 0.1173^*^ | 0.0414^*^ | 0.1022^*^ | 0.1788^*^ | 0.1464^*^ | 0.1560^*^ | 0.1023^*^ |
| YC_17 | 0.2253 | 0.2853 | 0.2587 | 0.2475 | 0.1892 | - | 0.2042^*^ | 0.2930^*^ | 0.1802^*^ | 0.1731^*^ | 0.2198^*^ | 0.1993^*^ | 0.1895^*^ | 0.3070^*^ |
| AD_17 | 0.2008 | 0.3462 | 0.1989 | 0.1900 | 0.1525 | 0.1983 | - | 0.2348^*^ | 0.1765^*^ | 0.2145^*^ | 0.3130^*^ | 0.0232^*^ | **0.0080**^NS^ | 0.2003^*^ |
| MY_17 | 0.0836 | 0.2622 | 0.0185 | 0.1873 | 0.0987 | 0.2590 | 0.2139 | - | 0.1521^*^ | 0.1211^*^ | 0.2368^*^ | 0.2252^*^ | 0.2456^*^ | 0.0898^*^ |
| BS_17 | 0.1196 | 0.2051 | 0.1320 | 0.1420 | 0.0380 | 0.1667 | 0.1713 | 0.1256 | - | 0.1150^*^ | 0.1680^*^ | 0.1621^*^ | 0.1757^*^ | 0.1198^*^ |
| JiJ_17 | 0.1186 | 0.1556 | 0.1013 | 0.2399 | 0.1016 | 0.1655 | 0.2160 | 0.1067 | 0.1142 | - | 0.0415^NS^ | 0.2130^*^ | 0.2212^*^ | 0.1270^*^ |
| IS_17 | 0.1611 | 0.1997 | 0.2173 | 0.3449 | 0.1638 | 0.2138 | 0.3039 | 0.2076 | 0.1589 | 0.0377 | - | 0.3055^*^ | 0.3159^*^ | 0.2201^*^ |
| JE_17 | 0.2024 | 0.3328 | 0.1997 | 0.1852 | 0.1432 | 0.1814 | 0.0258 | 0.2052 | 0.1541 | 0.2088 | 0.2893 | - | 0.0142^NS^ | 0.2043^*^ |
| GwJ_17 | 0.2198 | 0.3529 | 0.2199 | 0.1930 | 0.1578 | 0.1842 | **0.0109** | 0.2306 | 0.1712 | 0.2284 | 0.3120 | 0.0154 | - | 0.2204^*^ |
| JJ_17 | 0.0932 | 0.2359 | 0.0683 | 0.1882 | 0.0946 | 0.2714 | 0.1930 | 0.0660 | 0.1085 | 0.1144 | 0.1975 | 0.1937 | 0.2172 | - |

*: *P*<0.05 (significant value); NS: not significant.
