# Supplementary material for: Population Genetic Structure of Aphis gossypii Glover (Hemiptera: Aphididae) in Korea
Source: Insects. 2019 Sep 26;10(10):319. doi: 10.3390/insects10100319 (PMC6835795; doi:10.3390/insects10100319)
Supplement: Supplementary file 1 [file insects-10-00319-s001.zip › Supplementary Table 5.docx]

Supplementary Table 5. Pairwise *F*_ST_ ^[ENA]^ values (lower-left matrix), and pairwise *F*_ST_ values and significance (upper-right matrix) based on 8 microsatellite loci between the populations of *A. gossypii* in Korea (2018).

|  | HS_18 | CJu_18 | BS_18 | JE_18 | JJ_18 |
| --- | --- | --- | --- | --- | --- |
| HS_18 | - | 0.0277^NS^ | 0.0464^NS^ | 0.0639^*2^ | 0.1855^*^ |
| CJu_18 | 0.0290 | - | **0.0033**^NS^ | 0.1696^*^ | **0.2022**^*^ |
| BS_18 | 0.0448 | **0.0043** | - | 0.1862^*^ | 0.1964^*^ |
| JE_18 | 0.0659 | 0.1679 | 0.1804 | - | 0.1694^*^ |
| JJ_18 | 0.1719 | **0.2008** | 0.1945 | 0.1621 | - |

*: *P*<0.05 (significant value); NS: not significant.
